# Inflammation: the driver of poor outcomes among children with severe acute malnutrition?

**DOI:** 10.1093/nutrit/nuad030

**Published:** 2023-03-28

**Authors:** Jonathan P. Sturgeon, James M. Njunge, Claire D. Bourke, Gerard Bryan Gonzales, Ruairi C. Robertson, Mutsa Bwakura-Dangarembizi, James A. Berkley, Paul Kelly, Andrew J. Prendergast

**Affiliations:** Zvitambo Institute for Maternal and Child Health Research, Harare, Zimbabwe; Centre for Genomics and Child Health, Blizard Institute, Queen Mary University of London, London, UK; The Childhood Acute Illness & Nutrition Network, Nairobi, Kenya; KEMRI/Wellcome Trust Research Programme, Kilifi, Kenya; Zvitambo Institute for Maternal and Child Health Research, Harare, Zimbabwe; Centre for Genomics and Child Health, Blizard Institute, Queen Mary University of London, London, UK; Nutrition, Metabolism and Genomics Group, Division of Human Nutrition and Health, Wageningen University & Research, Wageningen, Netherlands; Zvitambo Institute for Maternal and Child Health Research, Harare, Zimbabwe; Centre for Genomics and Child Health, Blizard Institute, Queen Mary University of London, London, UK; Zvitambo Institute for Maternal and Child Health Research, Harare, Zimbabwe; The Childhood Acute Illness & Nutrition Network, Nairobi, Kenya; KEMRI/Wellcome Trust Research Programme, Kilifi, Kenya; Tropical Gastroenterology and Nutrition Group, University of Zambia, Lusaka, Zambia; Zvitambo Institute for Maternal and Child Health Research, Harare, Zimbabwe; Centre for Genomics and Child Health, Blizard Institute, Queen Mary University of London, London, UK

**Keywords:** inflammation, SAM, severe acute malnutrition, severe malnutrition, wasting

## Abstract

Severe acute malnutrition (SAM) is the most life-threatening form of undernutrition and underlies at least 10% of all deaths among children younger than 5 years in low-income countries. SAM is a complex, multisystem disease, with physiological perturbations observed in conjunction with the loss of lean mass, including structural and functional changes in many organ systems. Despite the high mortality burden, predominantly due to infections, the underlying pathogenic pathways remain poorly understood. Intestinal and systemic inflammation is heightened in children with SAM. Chronic inflammation and its consequent immunomodulation may explain the increased morbidity and mortality from infections in children with SAM, both during hospitalization and in the longer term after discharge. Recognition of the role of inflammation in SAM is critical in considering new therapeutic targets in this disease, which has not seen a transformational approach to treatment for several decades. This review highlights the central role of inflammation in the wide-ranging pathophysiology of SAM, as well as identifying potential interventions that have biological plausibility based on evidence from other inflammatory syndromes.

## Introduction

Severe acute malnutrition (SAM) is the most lifethreatening form of undernutrition and accounts for at least 10% of all deaths among children younger than 5 years in low- and middle-income countries.^1^ SAM is characterized by loss of lean muscle mass, fat mass, and/or the presence of edema. The World Health Organization (WHO) defines SAM in children aged 6–59 months as a weight-for-height *z* score of less than –3, mid-upper arm circumference <11.5 cm, and/or the presence of bilateral nutritional edema.^2^ The use of the word *acute* is intended to denote wasting or edema as processes that occur more quickly than stunting. In practice, the term reflects the frequent health deterioration that usually brings the child to medical attention, particularly in edematous SAM, which is frequently associated with acute infections. However, it is recognized that wasting has generally been present for a substantial period, and malnutrition is often chronic, given that almost all children with SAM have stunted growth, and half have severely stunted growth.^3^ Although we use the term *severe acute malnutrition* in this review for consistency with current WHO definitions, other authors use terms such as *severe malnutrition* or *severe wasting*. This highlights the heterogeneity and overlap of the populations involved, who may have sarcopenia, edema, and stunting. Differences between the acute wasting syndromes and the chronic stunting syndromes are listed in Table 1.^2,4–23^

Besides wasting with or without edema, SAM is a complex syndrome associated with defects in multiple organs, including the gut, liver, skin, and brain, and the immune, metabolic, and hormonal systems. Children who are admitted to hospital with complicated SAM (CSAM), due to severe edema, cognitive impairment (apathy, with suppressed appetite), or severe infection, have an inpatient mortality of 10%–40% in studies conducted in sub-Saharan Africa.^24^ Furthermore, it is increasingly clear that death is not restricted to the period of stabilization and initiation of therapeutic feeding in hospital. Follow-up of a recent cohort of children hospitalized for SAM in Zimbabwe and Zambia showed that the 1-year postdischarge mortality rate was 9.4%, which almost equaled the mortality rate seen in hospital.^13^ Similarly, among children aged 2–23 months who were hospitalized for acute illness at 9 sites in Africa and South Asia, the mortality rate was 11.2% in the 180 days after discharge in those with severe wasting or kwashiorkor, which was 7-fold higher than in well-nourished children.^25^

Where standardized WHO treatment guidelines for children with SAM^4^ are rigorously deployed, the majority of children do not die of causes directly related to low lean mass, such as hypoglycemia. Instead, studies show a predominance of infectious mortality, suggesting that the underlying pathophysiology of SAM includes abnormalities influencing functional immunity, including impairment of mucosal barriers, impaired cell energy metabolism,^26^ and systemic immune cell dysfunction.^27^ SAM is also associated with substantial morbidity: Children discharged from hospital after CSAM often continue to have growth impairments, cognitive defects, ongoing susceptibility to infection, and changes associated with long-term cardiovascular and metabolic disease.^15,28^ This suggests that unresolved multisystem pathological changes persist in SAM despite nutritional recovery and may be important drivers of the poor long-term outcomes in this vulnerable group.

Inflammation, whether systemic or local, is an evolutionarily conserved process characterized by the activation of immune and nonimmune cells that protect the host by eliminating cell debris and misplaced or altered host molecules, pathogens, and toxins, together with diversion of nutrients away from anabolic processes to host defense while promoting tissue repair and recovery.^29–31^ Emerging evidence suggests that both systemic and local inflammation may drive many of the physiological disturbances in SAM. Systemic inflammation is heightened in children with CSAM, and this has been shown to be independently associated with mortality^7^; however, because many children also have active infection, it is hard to isolate the degree of inflammation attributable solely to malnutrition processes. Care should be taken to extrapolate this to all children with uncomplicated SAM, because no comparable data on inflammatory markers are published from children with uncomplicated SAM.^8^

**Figure 1:**
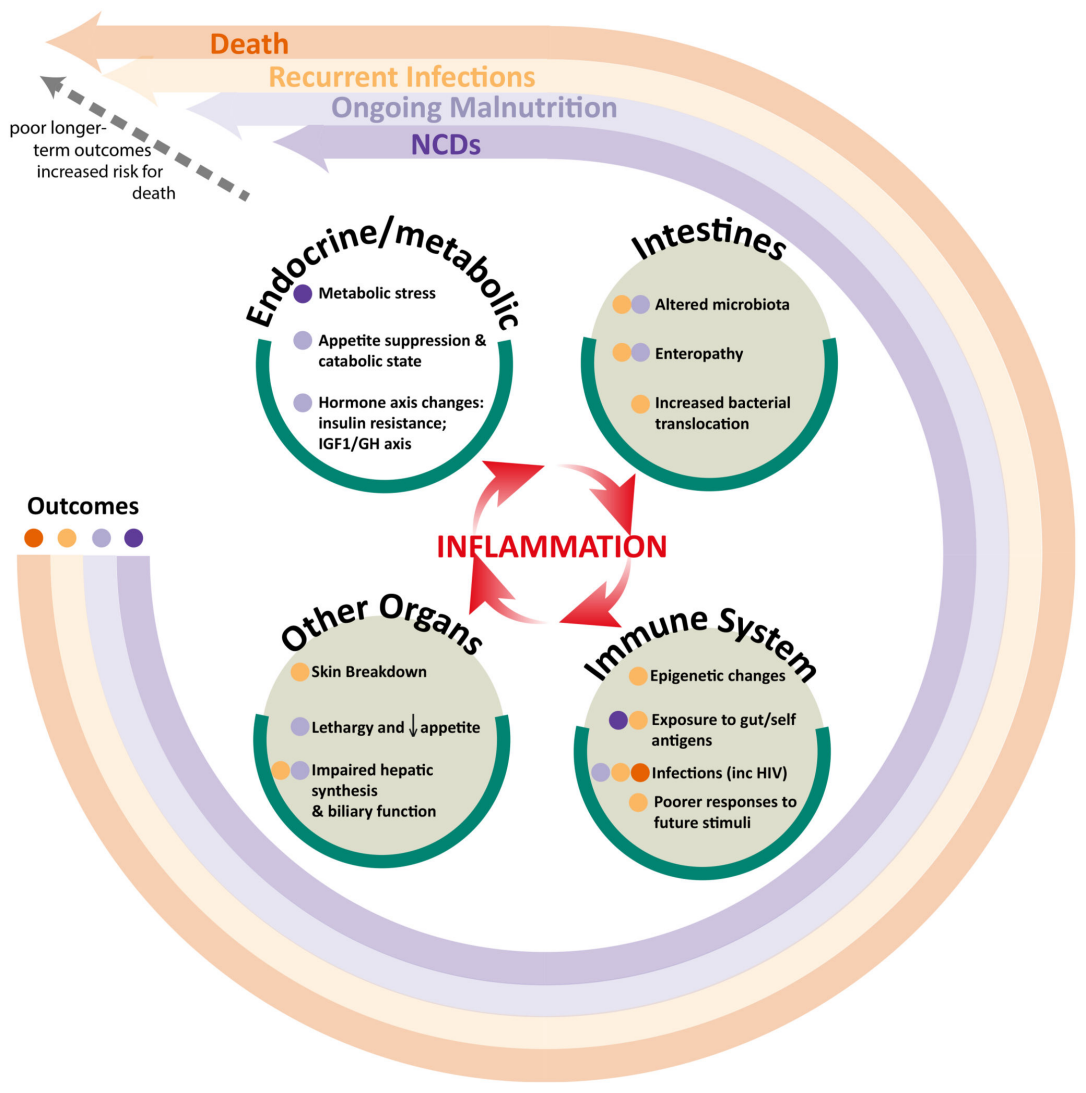
Conceptual framework showing inflammation as being central to driving the pathophysiology of the changes seen in severe acute malnutrition in the gut and other organs, and the immune system. These changes affect long-term outcomes, including nutritional state, infections, noncommunicable diseases, and death. Abbreviation: GH, growth hormone; inc, including.

In this review, we explore the impact of inflammation, and the drivers of inflammation, on the underlying physiological perturbations in children with SAM. We navigate the structural and functional changes in multiple organs, and in the immune, metabolic and endocrine systems. These pathophysiological pathways underlying SAM are viewed as both driving and resulting from inflammation (Figure 1).^32^ The persistence of inflammation and the drivers of inflammation in SAM, which mirror the pathophysiological pathways underlying other chronic diseases exhibiting a pro-inflammatory state such as chronic kidney disease, may be central processes mediating adverse long-term outcomes in SAM.

## Methods

Our primary objective for this review was to summarize the existing evidence that inflammation is implicated in the multisystem pathophysiology of SAM. A literature search was undertaken using the PubMed and Google Scholar databases, searching on the following terms: “severe acute malnutrition,” “severe malnutrition,” “severe wasting,” “marasmus,” and “kwashiorkor” for SAM papers; “enteropathy,” “malnutrition enteropathy,” and “gut” were added for the gut; “inflammation,” “inflammatory,” “immune system,” “innate immune,” and “immunosuppressed” for the immune system and inflammation; “sepsis,” “infections,” “pneumonia,” and “diarrhoea” for infections; and “dermatosis” and “skin” for the skin lesions; “endocrine,” “growth hormone,” “metabolic,” “anabolic,” and “catabolic” for metabolism. Search terms for long-term outcomes included “noncommunicable disease,” “metabolic syndrome,” “cardiovascular event,” and “blood pressure.” Alternative chronic conditions included chronic kidney disease and chronic liver disease, along with “malnutrition.” Analysis of reference lists and authors’ knowledge of related research were used to identify additional papers and evidence. Because of the heterogeneity between children included in studies, different definitions of malnutrition, and limited interrogative studies on underlying pathophysiological mechanisms of SAM, a narrative approach was chosen to summarize the topic.

## Inflammation and the Pathophysiological Changes in Severe Acute Malnutrition

### Gut physiology and function

In children with SAM, there is a relatively well-described constellation of changes in intestinal histology and physiology, termed *malnutrition enteropathy* (ME), and these changes are characterized by altered morphology and function of the gut. Alterations in the structure of the small intestine in SAM include reduction in villus height and number, and crypt hyperplasia, resulting in a reduced surface area for absorption.^33^ Enteropathic changes described in SAM also include disruption of the gut epithelium, which allows passage of luminal gut microbial contents to pass to the systemic circulation among children with stunting.^34^ Histological specimens from children with CSAM and persistent diarrhea, which affects approximately two-thirds of children with CSAM in southern Africa,^35^ demonstrate reduced expression of the tight junction protein claudin 4 and disrupted expression of E-cadherin, the adherens junctional protein, at sites of epithelial breaks compared with non-malnourished children with persistent diarrhea^17^ Coupled with the loss of goblet cells and a reduction in their mucus-producing capacity, the protective barrier function of the mucus layer is also impaired. Furthermore, there is a reduction in mucosal mass, and lower levels of IgA secretion in mucus.^36,37^ Proxies for epithelial integrity have also been deployed to characterize enteropathy in children with SAM. The ratio of passage of large vs small sugar molecules across the gut, such as the lactulose to mannitol ratio, is increased compared with community controls in some studies^20^ but not others.^17^ The larger of these sugars, lactulose, is still approximately a thirtieth of the size of lipopolysaccharide (LPS), the endotoxin found in the cell wall of gramnegative bacteria, making interpretation difficult. The exact method of increased transit of larger molecules such as LPS across the gut barrier is unclear; however, the visualization in adults of fluorescein “plumes” from sites of single-cell epithelial defects or erosions near the lateral intercellular space (corresponding to the expected position of tight junctions^38^) suggests a paracellular role. It remains to be shown if this definitively allows the passage of large molecules. Plasma LPS levels are markedly increased in children with CSAM^39^ and those with CSAM with protracted diarrhea,^17^ consistent with translocation of microbial products or even whole bacteria from the intestine. The presence of increased circulating LPS levels in a mouse model of moderate malnutrition suggests it may be part of the pathophysiology of SAM even in the absence of infection.^40^

These morphological and functional changes in the gut in children with SAM are accompanied by local enteric inflammation, which is characterized by infiltration of macrophages and lymphocytes (primarily CD3+ T cells) to the lamina propria and epithelium, with T-cell numbers increasing and B-cell numbers decreasing with worsening nutrition.^18^ Furthermore, the breaches in the gut barrier likely contribute to the heightened systemic inflammation observed in SAM.^37^ It is yet unclear whether the local and systemic inflammation that characterizes SAM is a cause or consequence of impaired gut barrier function; however, it is likely that these pathways are bidirectional.

The role of inflammation and an altered microbiome in the pathogenesis of enteropathy is also apparent in studies of environmental enteric dysfunction (EED; also termed environmental enteropathy). EED is generally milder than ME and is an asymptomatic small intestinal condition seen almost universally in children and adults in settings of impoverished low- and middle-income countries. EED results in similar architectural and inflammatory changes in the gut as ME,^41^ meaning the 2 are virtually indistinguishable macroscopically and likely overlap. EED is believed to be caused by chronic exposure to antigenic material, which is most likely due to recurrent enteropathogen acquisition in settings of poor sanitation and hygiene. It has also been hypothesized that specific ingested foods may cause an inflammatory response in the gut, in view of the weakly positive coeliac serology reported in children with SAM and diarrhea,^17^ or that micronutrient deficiencies or toxin exposure may be underlying causes. Differences and similarities between ME and EED are summarized in Table 1.^7,17–23^ EED may predispose a child to SAM through malabsorption in the context of an already marginal diet, together with chronic enteropathic changes; however, it is usually not lack of food alone that precipitates ME. Intestinal inflammation may drive more than just gut-specific effects: Enteropathy markers were also associated with longer-term linear growth outcomes^20^ and death^7^ in some studies, though not in others.^42^ Furthermore, once a child has established ME, refeeding only partially resolves the condition.^43^

The changes in the gut referred in children with SAM generally reflect alterations in the small intestine, which is amenable to histological examination after biopsy-specimen collection via endoscopy. This may well be the major site of gut pathology, given the key absorptive and endocrine functions of the small intestine. However, the majority of human SAM studies have not examined the role of the large intestine, despite the higher biomass of bacteria and consequent LPS load. Some studies have investigated fecal myeloperoxidase^7^ as a biomarker of intestinal inflammation, which is produced in the colon as well as the small intestine.^44^ An animal model of moderate acute malnutrition in mice revealed increased intestinal permeability in the cecum and colon,^40^ and an animal model of EED in primates revealed increased permeability in the colon, which was associated with growth faltering.^45^ These findings highlight the importance of additional studies conducted to examine the colon in children with SAM and the importance of animal models in helping to understanding the pathophysiology.

### Gut microbiota

Intestinal homeostasis relies on the interaction among the gut barrier, underlying immune system, and the commensal gut microbiota. Commensal microorganisms in the gut support normal metabolism, gut barrier integrity, immune function, and intestinal homeostasis through a variety of mechanisms. The diversity of the healthy gut microbiome also provides colonization resistance against enteropathogens by metabolic exclusion, and through direct antimicrobial antagonism, thereby reducing the risk of infection, with its consequential inflammation.^46^ Furthermore, the gut microbiome produces a plethora of metabolites, including short-chain fatty acids, which regulate inflammatory processes and induce subsets of regulatory T cells (specifically, ROR*γ*t T-regulatory cells) that also mediate intestinal homeostasis.^47^ The microbiota also contributes to the development of the immune system, with commensal-immune interactions shaping the maturation of CD4 T cells and IgA-producing plasma cells.^48^ Without normal gut antigenic stimulation, germ-free mice have impaired immune development^49^ and altered intestinal structure, with reduced surface area and a thinner epithelium.^50^ The relationship between the intestinal microbiota and the host immune system is bidirectional, suggesting that immune impairment and/ or inflammation may induce changes in the gut microbiota and vice versa. Studies from experimental mice report that impairment of T- and B-cell maturation fundamentally alters gut microbiota composition.^51^ Similarly, microbiota maturation was shown to be dependent on luminal *γ*-proteobacteria–specific host IgA responses in these experimental models.^52^ Mice with a knocked-out *Lyn* gene (a regulator of cell function and natural killer–cell activation after toll-like receptor stimulation) have increased intestinal damage–induced inflammation and altered microbiota.^53^

Evidence from animal studies suggests that altered gut microbiota in SAM drives inflammation and disturbed immune responses both locally, in the gut, and systemically. ME can be recapitulated in experimental mice by introducing a consortium of enteropathogens, but only in the presence of a low-protein, low-micronutrient diet.^54^ Even in the absence of overt pathogens, dysbiotic microbiome communities can induce malnutrition phenotypes and intestinal dysfunction, whereby a Bacteriodales and *Escherichia coli* consortium, in combination with a low-protein, low-micronutrient diet leads to impaired barrier function, intestinal inflammation, and an influx of *γδ*-T cells in the duodenum of experimental mice.^55^ This disturbed interaction also contributes to the proinflammatory intestinal microenvironment and SAM-like phenotype after fecal transfer from children with SAM into experimental mice.^55–57^ In addition to intestinal inflammation, these immune-microbiota interactions in SAM also include altered IgA-targeting of gut microbes.^58^ Therefore, in SAM, it is likely that a disturbed gut microbiota drives intestinal inflammation and vice versa.

In humans, a consistent microbiota signature of malnutrition is not apparent; however, it appears that uncomplicated SAM is associated with gut microbiota “immaturity” compared with healthy controls and is temporarily resolved during the use of ready-to-use therapeutic food.^59,60^ This has led to the development of gut-microbiota–directed complementary foods as alternatives to standard ready-to-use therapeutic foods (RUTFs), which aim to restore gut microbiota maturity in addition to providing host nutrients. A recent randomized trial in children with moderate acute malnutrition, which typically precedes SAM, found that a gut-microbiota–directed complementary formulation led to significantly greater growth recovery compared with standard RUTFs, despite lower calorie density, and was accompanied by greater “repair” of the gut microbiota.^61^

A number of pathways may contribute to the effect of the gut microbiota on inflammation in SAM. Short-chain fatty acids produced by particular microbial species are required for the proliferation of regulatory T cells, which can reduce some of the local intestinal inflammation that occurs after infection, as well as being 1 of the pathways involved in immunological tolerance.^62^ Furthermore, a healthy microbiota comprising mutualist bacteria can protect against enteric infection by suppressing enteropathogens through competition for nutrients, promoting production of antimicrobial peptides by epithelial cells and producing metabolites that affect the survival of pathogens.^18^ For example, the microbiota-activated PPAR-*γ* pathway prevents dysbiotic expansion by reducing the bioavailability of respiratory electron acceptors to Enterobacteriaceae (which includes *E. coli*) in the lumen of the colon.^63^ Reactive oxygen species produced by the host immune system after infection or injury can lead to outgrowth of Enterobacteriaceae because the bacteria use these agents for anaerobic respiration, which thereby excludes other fermenting bacteria.^64^ Randomized trials of probiotics in SAM have produced varying effects^65^ but may be able to reduce diarrheal incidence, suggesting restoration of intestinal homeostasis.^66^

### Enteric and systemic infections

Infections, including diarrhea and respiratory infections, are more common and more severe in children with SAM^67^ compared with healthy children,^68^ and deaths among children with SAM are predominantly due to infections.^69^ Children with SAM are at particularly high risk of enteric infections and enteric dysfunction. Although there are varying reports of the prevalence of diarrhea in children presenting to hospital with SAM, this can range from 40%^70^ to more than two-thirds in some settings.^35^ Diarrhea is both a cause and an effect of malnutrition, being a consequence of the morphological changes seen in ME, and diarrhea can result from a cytokine storm, such as in sepsis. Its prevalence and duration are increased in children with malnutrition,^71^ with more than two-thirds of children with CSAM presenting with diarrhea.^35^ Enteric infections have profound consequences: As well as increasing local and systemic inflammation through immune responses to the pathogen, infections reduce nutrient availability, due to intestinal malabsorption, increased metabolic needs, increased losses, and altered absorption.^72^ Pathogen–microbiome interactions, currently poorly understood, can augment this response with altered metabolism and storage of dietary calories as body fat.^73^ In a recent meta-analysis in children hospitalized with SAM, the presence of diarrhea was independently predictive of death.^12^

Enteropathogens including *E. coli*, *Shigella*, norovirus, and sapovirus had stronger associations with moderate-to-severe diarrhea in children with acute malnutrition than in better-nourished children serving as a control group.^74^ These pathogens can become asymptomatic and persistent because of impaired mucosal immune responses and, consequently, exacerbate malnutrition. In a study of children hospitalized with CSAM in Malawi, 55% still had an enteric pathogen detectable after stabilization,^75^ and there was some evidence for an association between weight-for-height *z* score and prolonged rotavirus shedding after immunization.^76^ The increased propensity for infection in SAM leads to more invasive infections with enteropathogens or pathobionts,^68,77^ with such infections being associated with poor outcomes. These processes also drive the ongoing cycle of inflammation and increased intestinal permeability.

Similarly systemic infections can both increase inflammation through immune responses to the pathogen^78^ and exacerbate malnutrition through reduced appetite and nutrient intake, increased nutrient loss, diversion of nutrients to inflammation, increased metabolic rate, and increased tissue catabolism. Pneumonia is a major cause of inpatient mortality both in children with CSAM and in those with less severe forms of malnutrition.^79^ The etiology and frequency of infectious organisms in children differ by nutritional status, with *Klebsiella* species and *Staphylococcus aureus* being the most common organisms in children with SAM.^79^ The risk of an invasive bacterial infection is significantly higher in those with SAM^80^ compared with well-nourished children, and children with SAM who had bacteriaemia were more likely to have bacteria traditionally seen as contaminants in well-nourished children (eg, coagulase-negative *Staphylococcus*) but may be considered pathogenic in children with SAM,^77^ although this study was small.

In a recent meta-analysis, 4.1%–51% of children presenting with SAM in sub-Saharan Africa had HIV infection,^12^ which is associated with a 4-fold higher inpatient and outpatient mortality rate compared with children with SAM alone, after adjusting for confounding factors.^13^ HIV interacts with SAM through multiple pathways, including direct HIV-mediated cellular injury to gut, kidney, and neuronal cells, together with indirect effects, including chronic immune activation and opportunistic infections arising from the immunosuppressed state.^81^ Studies of children without malnutrition show the loss of gut CD4^+^ T cells arising from HIV infection, which leads to epithelial injury, compounding the loss of mucosal integrity and leading to systemic inflammation, through microbial translocation across the gut.^82^ Further inflammation can lead to apoptosis and/or pyroptosis followed by necroptosis (ie, cell-death mechanisms triggered by inflammation) of uninfected cells,^83^ leading to both systemic inflammation and specific cell destruction, including that of neurons and kidney cells.^84^

Together, these indirect and direct processes reinforce the inflammation, enteropathy, and immune dysfunction cycle, with some arguing that HIV-SAM is pathophysiologically distinct from SAM alone and likely contributing to the higher mortality rate.^85^ Consistent with this, children with HIV and SAM have higher levels of inflammatory markers, including immunoglobulins, calprotectin, complement, and proteins related to the host response to infection, such as lipopolysaccharide binding protein, compared with children with SAM alone.^86^

### Immune system

Unlike the relatively well-described morphological changes seen in the gut, perturbations in the immune system are less well defined. Children with SAM broadly have increased levels of local and circulating proinflammatory cytokines compared with healthy children, but the exact soluble and cellular changes are poorly characterized. For example, Rytter et al^87^ found a heterogeneous profile between studies, with mostly consistent findings of increased levels of C-reactive protein but conflicting results with other immune biomarkers. Few studies to date have taken advantage of longitudinal interrogation using modern multichannel platforms that can concurrently analyze multiple immune markers from a small biological sample.

Authors of more-recent studies have reported that markers of systemic inflammation such as C-reactive protein, soluble CD14, TNF-a, and IL-6 are increased in children with CSAM compared with well-nourished children serving as controls.^7,88–90^ Although these changes may be anticipated, given that children frequently present with infections, these results do highlight that children with CSAM can mount a robust inflammatory response despite the shutdown of other metabolic systems.^78^ Despite very low whole-body protein levels, children manage to produce inflammatory and acute phase proteins, and it has been suggested that this may occur through different mechanisms than those in well-nourished children, with a decreased rate of catabolism of proteins playing an important part, in addition to protein synthesis.^91^ In a 2016 Malawian study, systemic inflammation (characterized by IL-1RA, IL-6, IL-2, TNF-*α*, TNF-*β*, and IL-13), was associated with death in children with CSAM, independently of intestinal inflammation.^7^ Similarly, a 2019 Kenyan study used proteomics in children with CSAM to show that upregulated inflammatory and endothelial function proteins were associated with death.^92^ Although many of these cytokines may be associated with neutrophil activation (a finding supported by increases in levels of other biomarkers, such as plasma calprotectin), the cytokine patterns reported do not identify a single innate or adaptive pathway driving inflammation. Many of these studies compare children with CSAM with children who are neither ill nor malnourished. Therefore, it is impossible to know whether the observed inflammation is caused by malnutrition or concurrent infection, or the interaction of both. There are likely to be multifactorial drivers of a persistent inflammatory environment, including systemic exposure to translocated gut bacterial products, ongoing bacterial infection, and deranged immune responses during nutritional recovery.^92^

**Figure 2:**
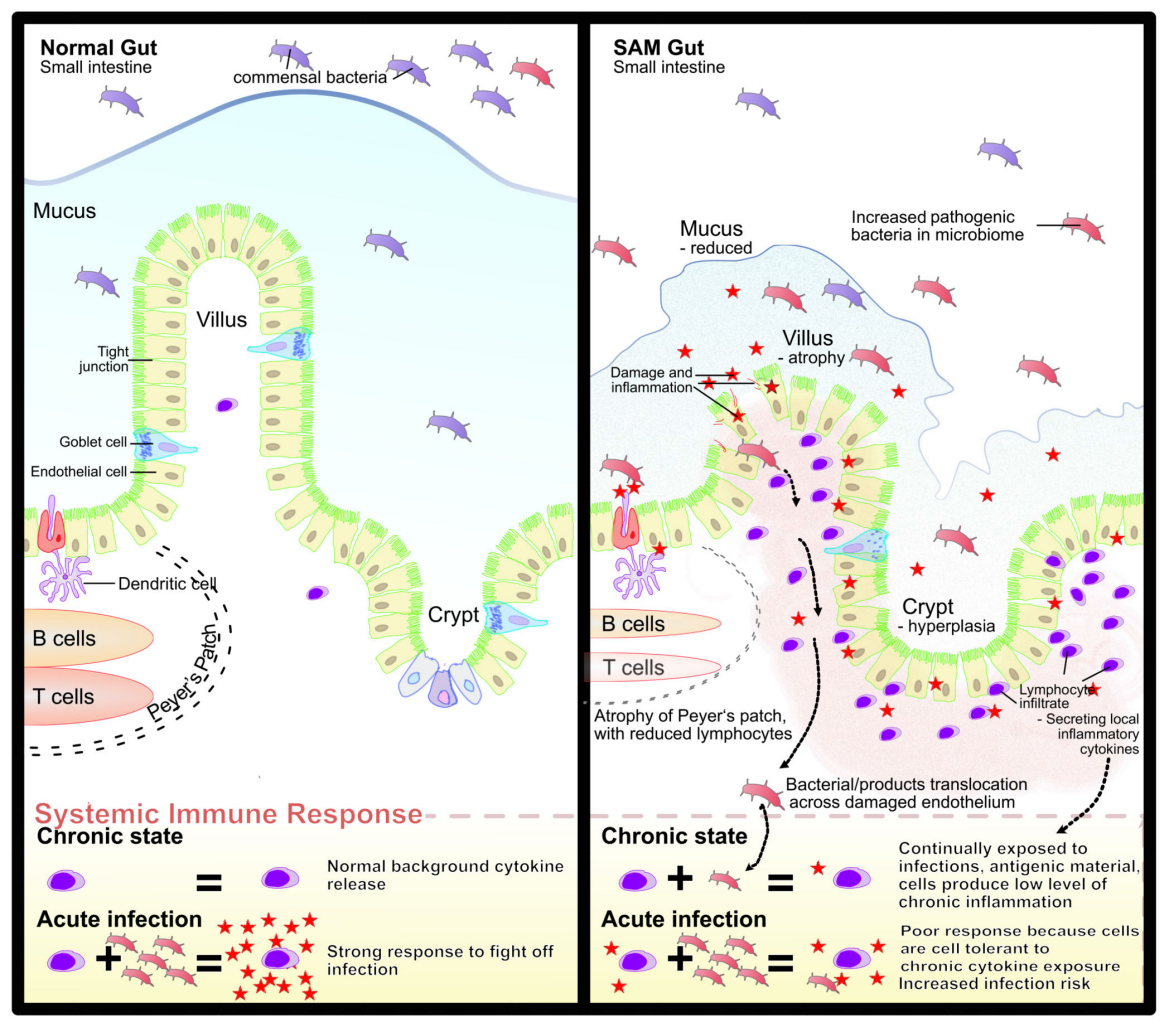
Depiction of the healthy gut (left) and changes seen in the small intestine of children with severe acute malnutrition SAM (right). Infection, altered microbiota, exposure to antigens, and translocation of bacterial products result in long-term level of chronic inflammation, which affects the body’s subsequent response to infections (bottom)

The effect of a proinflammatory environment on immune function can be profound, leaving the child more vulnerable to subsequent infections. Sudden or chronic exposure to immunogenic stimuli can lead to a refractory period of immune dysfunction or immunoparalysis.^93^ Exposure of monocytes in vitro to the bacterial cell wall component LPS provokes a strong cytokine response, but also alters responses to future stimuli through epigenetic remodeling of innate immune cells in a process called endotoxin tolerance.^94^ This has been demonstrated in children with CSAM in Zambia, where a study showed that dendritic cells, required for the generation of antigen-specific T-cell responses, were anergic in children with a high level of circulating LPS.^95^ This rendered dendritic cells less sensitive to endotoxin and more prone to generate IL-10, whose immunoregulatory properties may prevent the child from mounting a robust antipathogen response. This can be viewed as a reinforcing cycle, because the inflammatory response that occurs after exposure to LPS has itself been shown to increase intestinal permeability in animal models,^96,97^ permitting further translocation of LPS and bacterial products from the gut. Even children hospitalized with noncritical infections have increased gut permeability, endotoxemia, and altered monocyte phenotype and function.^98^ In children with SAM, who have a background state of antigenic stimulation leading to chronic inflammation complicated by severe systemic infections, this pathogenic cycle is likely to be more profound (Figure 2).

The consequence of immune dysfunction after an initial inflammatory insult is highlighted in a Kenyan study during which researchers followed children with pneumonia, after admission to hospital. This initial infection rendered children more susceptible to subsequent episodes of pneumonia over the next 24 months,^99^ as well as death.^100^ This phenomenon is similar to the increased infection risk seen in previously healthy children after admission to intensive care units for the management of sepsis.^93^ Children with CSAM have a particularly high mortality rate in the period after discharge from nutritional rehabilitation programs. The Follow-Up of Postdischarge Growth and Mortality After Treatment for SAM (FuSAM) study of 1024 children showed that 192 deaths in children admitted with CSAM occurred after treatment discharge, similar to the 238 deaths occurring in hospital, although a high proportion of children were HIV positive.^14^ Similarly, the recent Health Outcomes, Pathogenesis and Epidemiology of SAM (HOPE-SAM) study showed a mortality rate of 9.1% in the 48 weeks after discharge, matching the inpatient mortality rate observed,^13^ and the Childhood Acute Illness and Nutrition (CHAIN) study showed a 180-day postdischarge mortality rate of 11.2% in children with SAM, 7 times higher than well-nourished children.^25^ Although not fully characterized, because most deaths occurred in the community, these children predominantly had infective causes of death. A recent pooled analysis showed that diarrhea, respiratory illnesses, measles, and malaria are major causes of death.^101^ Taken together, these findings suggest a period of vulnerability to infection that persists after discharge from hospital and that needs to be addressed to prevent death from infectious disease during the protracted period of convalescence.

### Endocrine and metabolism changes

Multiple aspects of the endocrine system are altered in children with SAM. Studies report lower levels of pituitary-produced growth hormone and pancreatic insulin, as well as the more downstream mediator insulin-like growth factor 1, compared with levels in healthy children,^17,102^ with subsequent evidence of growth hormone tolerance and insulin resistance during recovery.^103^ Such changes likely mediate the longer-term sequelae of noncommunicable diseases (NCDs) seen in children with SAM, including a heightened risk of metabolic syndrome.^104^

Inflammation appears to play a crucial role in these endocrine changes in animal models. In mice, higher concentrations of the proinflammatory cytokine IL-6 lead to a decrease in IGF-1 concentrations, resulting in growth impairment.^105^ Among children with SAM in the Interactions of Malnutrition and Enteric Disease (MAL-ED) study, serum C-reactive protein levels were positively associated with growth hormone levels and negatively associated with IGF-1 and its principal binding protein, IGFBP-3, indicating a state of growth hormone resistance,^103^ which was associated with reduced height-for-age *z* scores. Similar associations between inflammation and long-term linear growth, through reduced IGF-1 and IGFBP3 levels, are seen in children with stunting.^5^

Studies of children with SAM have found impaired glucose uptake into cells, which is associated with pancreatic *β*-cell dysfunction.^106^ Animal models have shown that this effect is exacerbated by increased circulating endotoxin levels.^107^ Pancreatic glucose toxicity can be mediated by oxidative stress,^108^ and pancreatic exocrine function in children with CSAM has been linked to pancreatic inflammation.^109^ Taken together, these suggest that symptoms caused by inflammation and oxidative stress are reflected in children with SAM, indicating a potential an etiologic pathway for these metabolic changes.

The liver is usually altered in children with SAM, particularly among those with edema, with evidence of hepatomegaly, steatosis with fatty deposits, and vacuolation of hepatocytes, even in the absence of edema.^110,111^ Animal malnutrition models of liver dysfunction show that there is peroxisomal loss and dysfunction and impaired hepatic mitochondrial production of adenosine triphosphate.^111^ These changes have been compared with nonalcoholic fatty liver disease. Small intestinal bacterial overgrowth with translocation of LPS and subsequent transport along the portal vein is implicated in the development of fatty liver disease.^112^ The role of local inflammation in the liver in SAM is unclear, but chronic inflammation plays a key role in the progression of disease in nonalcoholic fatty liver disease.^113^

Presence of chronic inflammation compounds malnutrition because, in animal studies, it suppresses appetite and promotes a catabolic state. Animal models show that administration of proinflammatory cytokines such as IL-1*β* or stimuli such as LPS reduces meal frequency and size.^114^ Inflammation-induced anorexia is likely to be an active host defense strategy against serious infection, to help pathogen elimination.^115^ Such a mechanism in SAM, where malnutrition is chronic, can be seen as maladaptive: The inflammation is at least partly due to the malnutrition itself, and the presence of inflammation perpetuates the ongoing malnutrition. One study has suggested a potential evolutionary survival benefit to this ongoing inflammation in environmental enteropathy, being potentially reduced bacterial translocation (proxied by plasma LPS, lipopolysaccharide binding protein, and soluble CD14) at the cost of impaired growth.^116^

### Skin

Children with edematous SAM, especially edematous CSAM in hospital, can have profound dermatosis with skin erosions. The etiology of the lesions is unclear, but potential processes include specific amino acid deficiencies affecting collagen, micronutrient deficiencies, or essential fatty acid deficiencies.^117^ Increased levels of plasma extracellular matrix proteins were also found in children with edematous CSAM compared with none-dematous CSAM matched by lower serum albumin levels,^118^ which could also explain the loss of skin integrity. Extracellular matrix degradation is often associated with inflammation.^118^ Because dermatosis characteristically affects pressure points, areas of edema, and the perineum, erosion of the skin barrier in these locations constitutes an important point of entry for bacteria, where, in the context, of a functionally immunocompromised host, even commensal bacteria can act as invasive pathogens. The presence of dermatosis is an important predictor of treatment failure, because of increased risk of infections,^119^ further perpetuating inflammation.

### Long-term outcomes

Children with a range of early-life insults are at increased risk of NCDs through epigenetic modifications, which can occur from the periconception period through to adulthood.^120^ Children with low birth weight have increased risk of diabetes and cardiovascular disease later in life,^121^ and there is evidence that SAM also predisposes to adult chronic disease. One study of CSAM survivors (using former definitions of malnutrition), whose mean age was 28.8 years, found higher diastolic blood pressures and systemic vascular resistance compared with non-malnourished control participants, which led to a greater risk of hypertension in later life.^122^ In a 7-year CSAM follow-up study in Malawi, children surviving CSAM had patterns of “thrifty growth” associated with “future cardiovascular and metabolic disease,”^15^ although it should be noted that these children had a median age of 9.3 years, so the effect of CSAM on NCDs is uncertain. One systematic review found an increased risk of hypertension, impaired glucose metabolism, and metabolic syndrome after SAM and, in some cases, after severe famine.^28^ Inflammation is likely to play a fundamental role in these longer-term NCD outcomes because it has been implicated in both the formation of atherosclerotic changes^123^ and the poorer outcomes seen in type 1 diabetes, even after the normalization of glycemia.^124^

Children with SAM were also more likely to be stunted (height-for-age *z* score < –2) than well-nourished children in a study^15^; in the HOPE-SAM cohort, 50% of children with SAM had severely stunting (height-for-age *z* score < –3).^13^ Children with stunting, compared with those without stunting, have higher levels of inflammatory biomarkers, suggesting that stunting itself may be an inflammatory disorder.^5^ Children with stunting also have longer-term sequelae of inflammation, such as hypertension, cardiovascular disease, and type 2 diabetes.^125,126^ The overlapping anthropometric defects of wasting and stunting may interact to compound this long-term pathology, which impairs long-term health. There is also evidence that long-term survivors of SAM in the Democratic Republic of Congo had decreased cognitive function compared with community control participants.^127^

## Inflammation in Other Related Syndromes

Inflammation is a biologically plausible mediator of outcomes among children with SAM, driven by the multifaceted perturbation in physiological pathways. There are other syndromes arising in the context of chronic disease that have phenotypic and pathological overlap with SAM. Malnutrition–inflammation complex (or cachexia) syndrome is a condition seen in children with chronic kidney disease (CKD). In malnutrition–inflammation complex syndrome, a strong relationship is seen between increased levels of inflammatory markers, low albumin levels, and malnutrition, although the exact definition of malnutrition is not standardized. Patients with chronic inflammation have a reduced appetite, increased protein depletion in skeletal muscles, fat and muscle wasting, hypercatabolism, endothelial damage, and atherosclerosis.^128^ Collectively, these changes are associated with increased risk of cardiovascular disease and death over at least 5 years of follow-up.^129^

The gut appears to underlie some of the inflammatory processes in malnutrition–inflammation complex syndrome. In patients with CKD, there is increased intestinal permeability, which is both a consequence of CKD and a contributor to its progression.^130^ Disruption of tight junction integrity arising from depletion of occludin, claudin, and zonula occludens through a post-translational mechanism leads to increased intestinal edema, monocyte infiltration, and permeability.^131,132^ Increased bacterial translocation may explain the endotoxemia seen in end-stage renal failure. This process, which generates chronic inflammation and associated immune modulation, means patients with end-stage renal failure have a similarly elevated infection risk as individuals with immunodeficiency or who are receiving immunosuppressive therapy.^133^ A similar functional immunosuppression is seen in people with cirrhosis, in whom, in addition to the failure of hepatic synthetic production of immune components, there is increased gut permeability and endotoxemia secondary to portal hypertension^134^; the ensuing chronic inflammation is also a necessary part of the pathogenesis of hepatic encephalopathy.^135^ The role of the inflammatory processes in these diseases, and the treatment approaches used, can provide insights into potential therapeutic targets in SAM.

### Interventions targeting the inflammation–enteropathy–immune dysfunction axis

**Figure 3:**
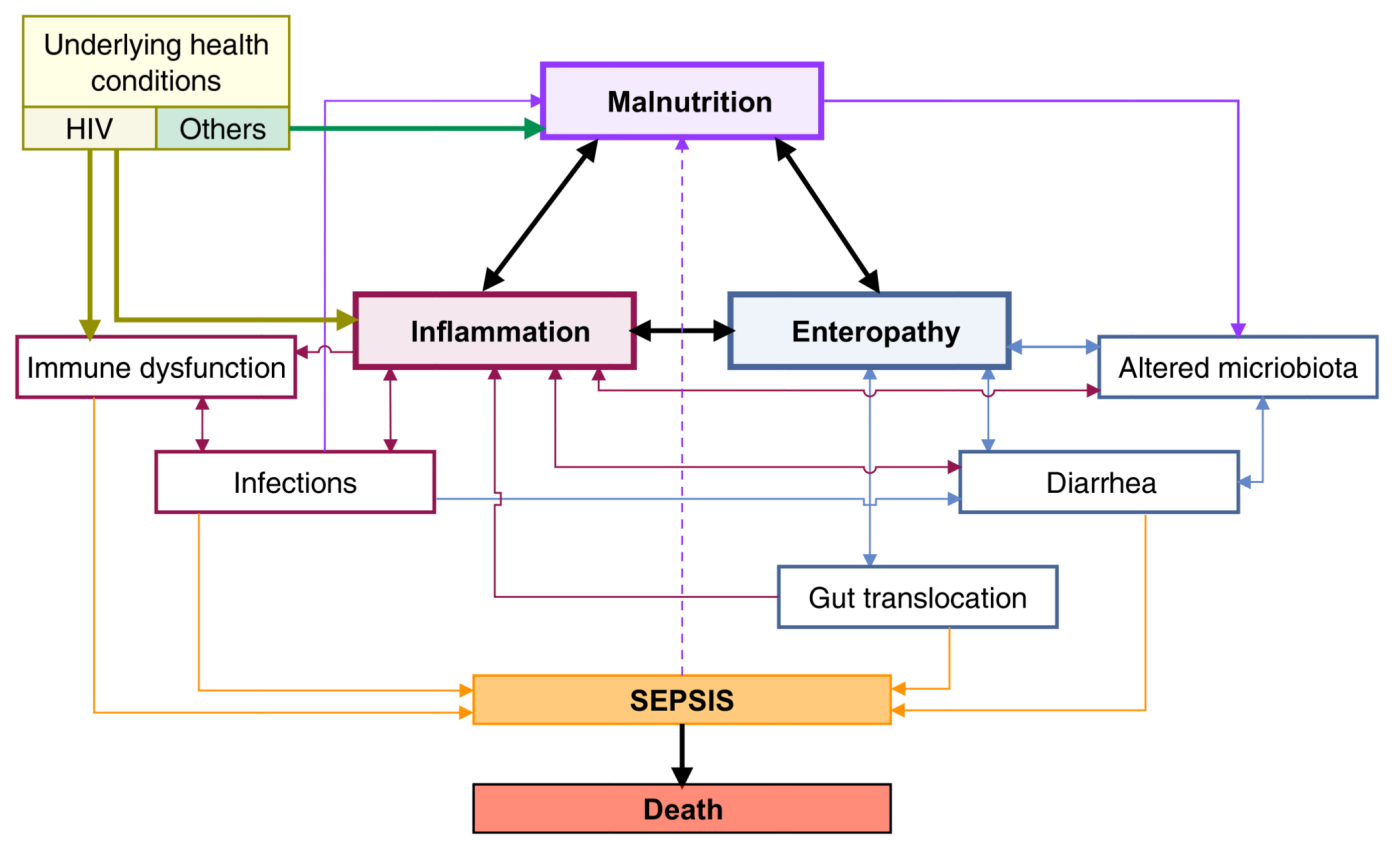
Conceptual framework of the interactions among enteropathy, gut translocation, and inflammation. There is a circular reinforcing response. Systemic inflammation can increase enteropathy and gut permeability, which, in turn, can increase gut translocation and expose the immune system to highly stimulating products

Despite evidence that infections and inflammation drive poor outcomes in SAM, few novel therapeutics targeting this pathway have been evaluated to date, beyond the use of antibiotics to treat underlying infections.^136,137^ There is a dearth of clinical trial data on interventions in children with SAM that explicitly target the interactions shown in the conceptual framework in Figure 3.

Broad-spectrum antibiotics are routinely recommended for children hospitalized with CSAM, although it should be noted that, due to antimicrobial resistance, adequate coverage of some organisms, such as members of the Enterobacteriaceae, is decreasing.^138^ Among children with uncomplicated SAM who are undergoing community-based nutritional rehabilitation, antibiotics have similarly been routinely recommended in guidelines, but the evidence base was poor until 2 recent trials. A double-blind trial in Malawi comparing a 7-day oral antibiotic course vs placebo showed significant benefits for nutritional recovery and mortality in a population of children with SAM with a high prevalence of HIV and a predominance of edematous malnutrition.^139^ Although these findings were not replicated in Niger, this study was underpowered to investigate mortality. There was a lower burden of HIV and kwashiorkor as well as a lower mortality rate, so benefits were difficult to determine; there was a reduction in referrals for hospital care, supporting the benefits reported in Malawi.^140^ It is unclear if the antimicrobial benefits in the Malawian trial arose from treatment of latent infections, which may be difficult to detect, or from reductions in inflammatory responses either directly through anti-inflammatory properties of the antibiotic itself^141^ or indirectly through treatment of co-infections, subclinical pathogen carriage, or remodeling of the gut microbiota. By contrast, a longer-term trial of 6 months of daily co-trimoxazole in HIV-negative children who had been hospitalized for CSAM did not show mortality reduction.^142^

Among children with HIV, management of SAM is currently similar to that of children without HIV, but there is a recognized need for new approaches, given the 4-fold higher mortality rate.^85^ Early treatment of HIV, if children are not already taking antiretroviral therapy, is recommended,^69^ although it is notable that half of children with HIV and SAM in a recent study from Zambia and Zimbabwe were not receiving antiretroviral therapy at the time of discharge from hospital.^13^ There is often a reluctance to start antiretroviral therapy in hospitalized children with advanced HIV and multiple co-infections, because of concerns about overlapping toxicity of medications, poor adherence, and immune reconstitution inflammatory syndrome; however, trial data show that rapid initiation is feasible and safe.^143^ Children with HIV should additionally start co-trimoxazole and isoniazid prophylaxis (for those older than 1 year and without tuberculosis), together with routine interventions depending on local needs (eg, deworming, malaria prophylaxis, vitamin A supplementation, growth monitoring). However, there is a need to identify additional strategies to reduce infections in children with HIV and SAM. In older children and adults with advanced HIV disease, a broader package of antimicrobial prophylaxis, targeting bacterial infections, tuberculosis, cryptococcal disease, and helminths reduced the mortality rate by 27%, and this strategy should be evaluated in younger children with HIV and SAM.^144^

Few other trials have evaluated interventions targeting inflammation and enteropathy in SAM. The Probiotics and Prebiotics for Severe Acute Malnutrition (PRONUT) study evaluated the role of a combined probiotic and prebiotic (“symbiotic”) treatment vs placebo in children with SAM in Malawi. The authors hypothesized that the formulation could improve immune function and gut microbiota dysbiosis and reduce pathogen carriage and associated inflammation.^65^ The results showed similar outcomes between arms, but there was some evidence of reduced outpatient mortality in the synbiotic group. A small Bangladeshi study with 59 children in each arm showed an improvement in weight-for-length *z* score, using microbiota-directed complementary food over RUTFs.^61^ The aminosalicylate mesalazine, an anti-inflammatory medication that decreases synthesis of prostaglandins and is used in the management of inflammatory bowel disease, was shown to be safe in a small proof-of-concept study,^89^ with biomarkers suggesting some evidence of benefit. A small pilot trial among children with uncomplicated SAM showed that RUTFs with an altered lipid profile (ie, with increased levels of the anti-inflammatory n-3 polyunsaturated fatty acid) was safe, and the study provided a strong foundation for future research.^145^ A small Malawian trial of pancreatic enzymes aimed at improving malabsorption and growth unexpectedly showed a mortality benefit, perhaps due to reducing small intestinal overgrowth^146^; another trial called Pancreatic Enzymes and Bile acids in acutely ill SAM [PB-SAM], is currently underway. A phase 2 trial of interventions aimed at ameliorating malnutrition enteropathy has just completed enrolment in Zimbabwe and Zambia. This trial evaluated 4 interventions, *N*-acetylglucosamine, colostrum, teduglutide and budesonide, all aimed at restoring intestinal barrier function^147^ and (for budesonide) reducing enteral inflammation, particularly in the small intestine.

Some of the therapeutic approaches from similar inflammatory-malnutrition conditions arising from other pathologies such as CKD and liver disease, and agents early in the therapeutic pipeline in SAM, are shown in Table 2.^66,69,89,145–166^ Other approaches have not yet demonstrated a clearly advantageous risk–benefit profile in other conditions; however, they provide plausible avenues for exploration as proof-of-concept interventions in children with SAM. A problem here is still the tendency to translate from other conditions, rather than developing de novo interventions for SAM, highlighting the lack of research into novel interventions for a neglected condition with a high global mortality rate.

## Conclusions

Inflammation appears to play a central role in the pathogenesis of SAM. The exact picture is not as clear as it could be, however, because many studies evaluate immune and inflammatory markers in children with CSAM compared with healthy control groups. Therefore, it is unclear which changes can be attributed to malnutrition alone and which are due to concurrent infection during the acute deterioration that brought the child to medical attention. Studies are needed to compare children who have CSAM with children who have uncomplicated SAM without acute infections.

Anti-inflammatory treatments are not currently part of the package of interventions provided to children with SAM. With the introduction of the 10-step WHO treatment guidelines for children with SAM in 1999, the aim was to reduce mortality from complications that are immediately life threatening. Although these guidelines were adapted subsequently, particularly with respect to the significant interplay between HIV and SAM, there have been no paradigm shifts in treatments for children with CSAM for the past 20 years. The mortality rate remains unacceptably high, with a recent systematic review showing an average inpatient mortality rate of 15.7% from studies published since 2000, despite adherence to guidelines.^12^ This is matched by a similar mortality rate in the year after discharge, highlighting the need for new inpatient management approaches and a focus on improving ongoing convalescent care.

Treatment of SAM has focused on the provision of nutrition, prevention of infection, and the avoidance of immediate consequences of pathophysiological processes such as hypoglycemia. These are highly effective when implemented well, such as the introduction of antibiotics to the outpatient management of SAM^139^; however, it is becoming increasingly clear there is a need to alter the trajectory of the underlying pathological processes in SAM to allow children to maximize their long-term potential. This need highlights the challenge in focusing only on the acute aspects of SAM, given the longer-term needs. Novel treatments targeting the inflammation associated with SAM, such as the trial of the safety of mesalazine,^89^ and new therapeutic approaches targeting malnutrition enteropathy^147^ show encouraging movement toward modifying underlying pathogenic pathways. Given the complex, multisystemic nature of SAM, it is likely to require multifaceted treatment approaches, or interventions that affect multiple different pathways of disease. Harnessing the full range of potential treatments for children with SAM is imperative in the global goal to end malnutrition in all its forms.^167^

## Figures and Tables

**Table 1:** The differences between malnutrition traditionally seen as more chronic (stunting) and that seen as more acute (wasting), and enteropathies seen in children

Classification	Stunting (traditionally considered more chronic)^a,b^		Wasting (traditionally considered more acute)^b,c,d^		
	Severe stunting	Moderate stunting	Moderate acute malnutrition	Uncomplicated SAM	Complicated SAM
Malnutrition					
Diagnosis	HAZ ≤ –3	HAZ between –2 and –3	Clinically well, with appetite, and at least 1 of the following^2^: MUAC < 12.5 cm; > 11.5 cm; WHZ between –2 and –3	At least 1 of the following^2^: WHZ ≤ –3; MUAC ≤ 11.5 cm; or bilateral nutritional edema	SAM with at least 1 of the following^2^: reduced consciousness/↓ appetite, infection, severe edema, or dehydration
Treatment	None currently		Community-based^4^		Inpatient management
Inflammation	Higher level of inflammation associated with increased stunting in Zimbabwean infants^5^		Higher levels of inflammatory markers^6^	Unknown	Higher systemic (and gut) inflammation^7,8^; associated with outcomes^7^ and linear growth^8^
Outcomes	Longer term: poorer cognitive function^5^; poorer economic productivity^5^; increased risk of infections and morbidity^9^		Shorter term: Deterioration if untreated.^10^ Lower risk of infection and death with moderate malnutrition. Longer term: Increasing risk of respiratory and diarrheal infections, and of death with lower WHZ,^9^ as well as relapse of malnutrition^11^		Shorter term: high inpatient mortality rate^12^ Longer term: high mortality rate (9.1%) over 1 y after discharge^13^; ongoing malnutrition,^14^ “thrifty” growth^15^
Enteropthies	*Environmental Enteric Dysfunction*		*Malnutrition Enteropathy^d^*		
Condition	Mucosal damage observed in children and adults in low-income countries		More severe mucosal damage seen in children with SAM		
Histologic changes	Villus blunting, crypt hyperplasia, inflammatory cell buildup in the intestines		Same as EED^17^; infiltration of CD3+ T cells; increasing numbers of T cells and decreased numbers of B cells with increasing malnutrition^18^		
Functional changes	Poor absorption of food, vitamins, and minerals		In addition to EED: autoantibody generation,^17^ altered microbiota		
Symptoms	Minimal or absent^19^		Frequently diarrhea (both infective and noninfective or inflammatory)		
Inflammation	Linear growth is lower in those with more gut inflammation.^20^		Greater, with gut inflammation associated with death^7^		
Outcomes	Longer term: malnutrition,^21^ anemia,^22^ growth stunting,^23^ impaired neurological outcomes^22^		Shorter term: LPS and inflammatory markers associated with intestinal permeability.^17^ Likely increased sepsis from translocation of intestinal bacteria Longer term: Linear growth associated with malnutrition markers		

a Loss of linear growth.

b Stunting and wasting have degrees of severity, with moderate and acute criteria shown in the table. Severe wasting (ie, severe acute malnutrition) can be further divided in to complicated and uncomplicated based on the additional symptoms or comorbidities. The enteropathies include environmental enteric dysfunction which is frequently asymptomatic, and the enteropathy attributable to severe acute malnutrition, which is frequently more severe.

c Loss of muscle and fat mass.

d High overlap of children with SAM and stunting.^16^

*Abbreviations:* EED, environmental enteric dysfunction; HAZ, height-for-age *z* score; LPS, lipopolysaccharide; ME, malnutrition enteropathy; MUAC, mid-upper arm circumference; SAM, severe acute malnutrition; WHZ, weight-for-height *z* score.

**Table 2:** Potential therapeutic interventions targeting inflammation, immune function, gut barrier function, and/or microbiota in SAM

Treatment	Therapeutic target	Notes^a^
Glucocorticoids	Anti-inflammatory, immune function	Useful in acute on chronic liver failure; detrimental with long-term use, especially for growth. Budesonide currently being trialed in SAM.^148^
Statins	Anti-inflammatory	Anti-inflammatory effect in adults with CKD^149^ but not trialed in children with CKD
Antioxidant vitamins, micronutrients	Anti-inflammatory	Vitamin E may improve outcomes in some studies of adults with CKD.^150^
Lipid content of ready-to-use therapeutic feed	Anti-inflammatory	Higher content of the anti-inflammatory n-3 poly-unsaturated fatty acid; trialed in uncomplicated SAM^146^
Aminosalicylate	Anti-inflammatory, barrier function	Showed safety and some evidence of biomarker improvement in children with SAM^89^
Albumin (intravenous)	Immune function	Low, due to poor synthesis, inflammation, and enteral protein loss. Can help restore innate immune function and reduce endothelial inflammation in cirrhosis.^151,152^ Restoration of immune function is separate from the controversial debate on intravenous albumin in kwashiorkor for low oncotic pressure.^153^
*N*-acetylcysteine	Immune function	No difference in preventing kwashiorkor in a blinded trial of 2372 children in Malawi^154^
Antibiotics	Immune function, infection/sepsis	Reduced mortality rate in CSAM. Long-term prophylaxis with co-trimoxazole had no mortality benefit in HIV-negative children with SAM. There are high resistance rates in children with SAM (community managed)^155^ and those admitted with CSAM,^69^ necessitating alternative antibiotics.
Immunization	Immune function, infection, sepsis	Inflammation may be driven by infection in CSAM. Reducing the incidence of vaccine-preventable diseases may be beneficial, while recognizing the efficacy of vaccines may be reduced in malnutrition.^156^
Granulocyte colony-stimulating factor	Immune function	Helps preserve neutrophil function in CKD^157^
Stem cell therapy	Immune function	Suppresses oxidative stress and inflammatory response in CKD^158^
MERTK inhibitor	Immune function	MerTK macrophages mediate liver damage in cirrhosis.^159^
*β*-blockers	Barrier function,	Decrease gut permeability and microbial translocation in patients with cirrhosis, possibly related to reduced portal hypertension^160^
Bile acids with or without pancreatic enzymes	Barrier function, inflammation	Modulate tight junction and barrier function in in vitro models.^161^ Pancreatic enzymes reduced mortality rate, potentially due to altering levels of protective bacteria.^147^
Rifaximin	Barrier function, Gut microbiota, infection	Trialed without positive benefit for 7 d in environmental enteropathy^162^ but not SAM.
Probiotics	Gut microbiota, barrier function	Trialed in SAM with diarrhea without effect on days of diarrhea,^163^ but has shown restitution of the gut barrier in Ugandan children with SAM, reducing incidence of diarrhea^66^
Fecal transplant	Gut microbiota, barrier function	Used in *Clostridium difficile* infections, trialed in cirrhosis.^164^ Not yet used in SAM, but has profound effect in animal models^165^
Glucagon-like peptide-2	Barrier function	Used in small-bowel syndrome promoting mucosal growth. Currently in a phase 2 trial in SAM^148^
*N*-acetyl glucosamine	Barrier function	Present on every cell surface. Supplementation restores the gut epithelial barrier in Crohn’s disease.^166^ Being evaluated in phase 2 trial in SAM^148^
Colostrum (bovine)	Barrier function	Promoter of mucosal healing.^167^ Being evaluated in phase 2 trial in SAM^148^

a Previous use and trials in either malnutrition or other conditions characterized by undernutrition and inflammation are listed.

*Abbreviations:* CKD, chronic kidney disease; CSAM, complicated severe acute malnutrition; SAM, severe acute malnutrition.
